# Molecular Control of Triplet-Pair Spin Polarization and Its Optoelectronic Magnetic Resonance Probes

**DOI:** 10.1021/acs.accounts.3c00556

**Published:** 2023-12-16

**Authors:** Obadiah G. Reid, Justin C. Johnson, Joel D. Eaves, Niels H. Damrauer, John E. Anthony

**Affiliations:** † National Renewable Energy Laboratory, Chemistry and Nanoscience Center, Golden, Colorado 80401, United States; ‡ Renewable and Sustainable Energy Institute, Boulder, Colorado 80309, United States; ¶ Department of Chemistry, University of Colorado Boulder, Boulder, Colorado 80309, United States; § Department of Chemistry, University of Kentucky, Lexington, Kentucky 40506, United States

## Abstract

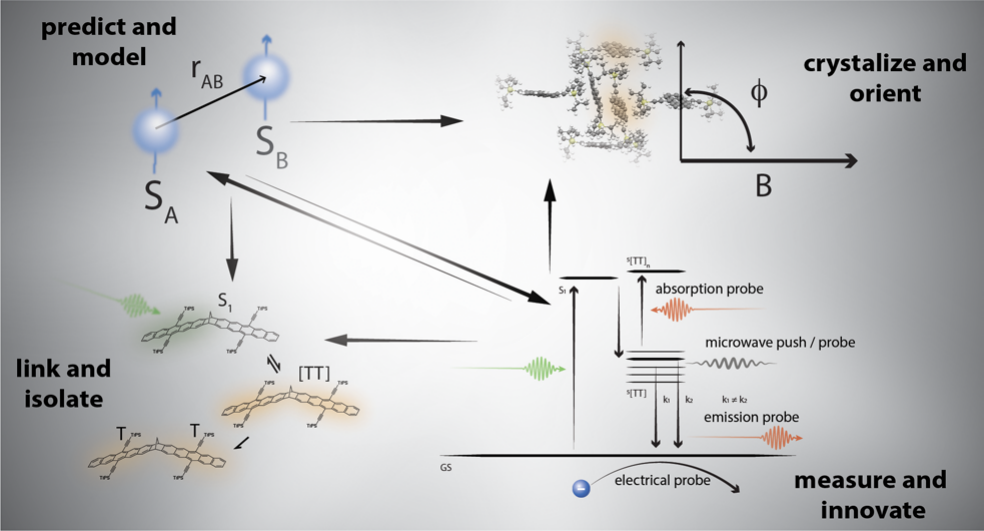

Preparing and manipulating pure magnetic states in molecular systems are the key initial requirements for harnessing the power of synthetic chemistry to drive practical quantum sensing and computing technologies. One route for achieving the requisite higher spin states in organic systems exploits the phenomenon of singlet fission, which produces pairs of triplet excited states from initially photoexcited singlets in molecular assemblies with multiple chromophores. The resulting spin states are characterized by total spin (quintet, triplet, or singlet) and its projection onto a specified molecular or magnetic field axis. These excited states are typically highly polarized but exhibit an impure spin population pattern. Herein, we report the prediction and experimental verification of molecular design rules that drive the population of a single pure magnetic state and describe the progress toward its experimental realization.

A vital feature of this work is the close partnership among theory, chemical synthesis, and spectroscopy. We begin by presenting our theoretical framework for understanding spin manifold interconversion in singlet fission systems. This theory makes specific testable predictions about the intermolecular structure and orientation relative to an external magnetic field that should lead to pure magnetic state preparation and provides a powerful tool for interpreting magnetic spectra. We then test these predictions through detailed magnetic spectroscopy experiments on a series of new molecular architectures that meet one or more of the identified structural criteria. Many of these architectures rely on the synthesis of molecules with features unique to this effort: rigid bridges between chromophores in dimers, heteroacenes with tailored singlet/triplet-pair energy level matching, or side-group engineering to produce specific crystal structures. The spin evolution of these systems is revealed through our application and development of several magnetic resonance methods, each of which has different sensitivities and relevance in environments relevant to quantum applications.

Our theoretical predictions prove to be remarkably consistent with our experimental results, though experimentally meeting all the structural prescriptions demanded by theory for true pure-state preparation remains a challenge. Our magnetic spectra agree with our model of triplet-pair behavior, including funneling of the population to the *m*_s_ = 0 magnetic sublevel of the quintet under specified conditions in dimers and crystals, showing that this phenomenon is subject to control through molecular design. Moreover, our demonstration of novel and/or highly sensitive detection mechanisms of spin states in singlet fission systems, including photoluminescence (PL), photoinduced absorption (PA), and magnetoconductance (MC), points the way toward both a deeper understanding of how these systems evolve and technologically feasible routes toward experiments at the single-molecule quantum limit that are desirable for computational applications.

## Key References

- SmyserK. E.; EavesJ. D.Singlet fission for quantum information and quantum computing: the parallel JDE model. Sci. Rep.2020, 10, 1848033116218 10.1038/s41598-020-75459-xPMC7595132.^1^ The foundational principles of triplet-pair spin polarization are developed through the analysis of a model spin Hamiltonian that highlights the role of molecular symmetries.
- RuggB. K.; SmyserK. E.; FluegelB.; ChangC. H.; ThorleyK. J.; ParkinS.; AnthonyJ. E.; EavesJ. D.; JohnsonJ. C.Triplet-pair spin signatures from macroscopically aligned heteroacenes in an oriented single crystal. P. Natl. Acad. Sci. U.S.A.2022, 119, e220187911910.1073/pnas.2201879119PMC930399035858318.^2^ A single crystal of a heteroacene is oriented and probed with time-resolved electron paramagnetic resonance (EPR), revealing the orientation-dependent population of important triplet-pair spin sublevels.
- DillR. D.; SmyserK. E.; RuggB. K.; DamrauerN. H.; EavesJ. D.Entangled spin-polarized excitons from singlet fission in a rigid dimer. Nat. Commun.2023, 14, 118036859382 10.1038/s41467-023-36529-6PMC9977721.^3^ A covalent dimer of pentacene with a rigid bridge is synthesized and shown to maintain remarkable spin polarization and coherence, revealed through time-resolved EPR techniques and theory.
- JoshiG.; DillR. D.; ThorleyK. J.; AnthonyJ. E.; ReidO. G.; JohnsonJ. C.Optical readout of singlet fission biexcitons in a heteroacene with photoluminescence detected magnetic resonance. J. Chem. Phys.2022, 157, 16470236319433 10.1063/5.0103662.^4^ Promoting transitions from the ^5^TT_0_ state is found to lead to a modulation in fluorescence from a heteroacene crystal, suggesting an optical readout that is distinct in character from that of free triplets.

## 1 Introduction

Qubit candidates abound in molecular systems, from magnetic nuclei^5,6^ to stable radicals,^7^ electronic excited states,^8^ and even anharmonic vibrations.^9^ However, each of these quantum degrees of freedom exhibits its own peculiar strengths and weaknesses, a situation ripe for designing hybrid systems that harness molecular engineering to take the best properties of each. For example, magnetic nuclei have extremely long spin coherence times, but their small gyromagnetic ratio couples weakly to electromagnetic fields, making gate operation times very long and leading them to require massive magnetic fields to obtain appreciable Zeeman splitting. The small energy scale inevitably leads to Boltzmann distributions that make the preparation of an initial pure quantum state exceedingly difficult. In electron spin systems, pure magnetic states are primarily prepared by going to ultralow (millikelvin) temperatures. At the other end of the spectrum, electronic excited states have the great advantage of large energy splitting and strong coupling to electromagnetic fields, making it trivial to prepare pure quantum states at room temperature; however, their decoherence processes are so fast that computing applications that harness them directly might be very difficult to implement.

**Figure 1:**
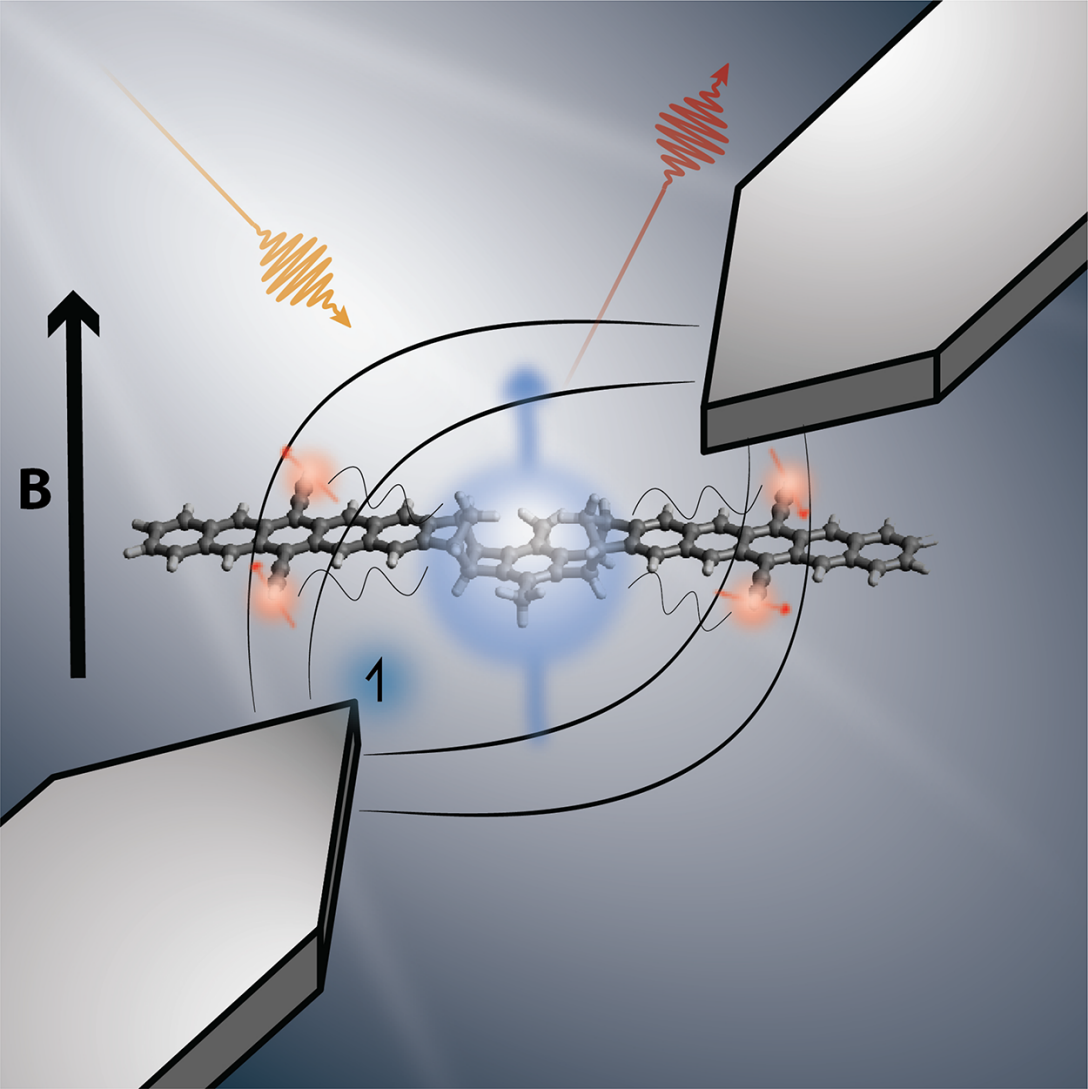
A futuristic vision of a singlet fission dimer situated in a molecular tunnel junction, allowing pure magnetic state preparation via optical excitation, coupling proximal magnetic nuclei into a molecular qubit network that can be read out either optically via emission probability or electrically via electron transmission probability.

Imagine instead a system that uses electronic excited states to prepare pure initial electron spin populations at high temperatures: states that can be used to map spin polarization onto proximal magnetic nuclei and mediate gate operations between them; states that facilitate both electrical and optical readout pathways toward the ultimate quantum limit of single-molecule qubit networks. Figure 1 illustrates such a system, showing a cartoon diagram of a singlet fission dimer situated in a molecular tunnel junction with both electrical and optical readout processes, along with couplings to specifically installed magnetic nuclei.

Although this vision is hugely optimistic, much of this functionality has already been demonstrated, either discretely in molecular systems^10−17^ or nearly all together in nitrogen vacancy centers in diamond.^18,19^ The challenge we have begun to address is developing the toolbox containing both the theoretical and chemical knowledge required to design and prepare hybrid qubit systems made to order that can be synthesized, assembled, and used at scale.

There are at least four photochemical pathways toward the vision articulated above: photoinitiated radical ion pairs,^14,15,17,20^ ligand-field state engineering,^21^ photoexcited triplets derived from intersystem crossing,^11,12^ and singlet fission (SF).^2−4,16,22−25^ Our work has focused on the last of these pathways due to its unique and potentially powerful properties, including ultrafast (sub-nanosecond) state preparation, the possibility of controlling spin evolution through both the inter- and intramolecular structure, and the demonstrated optical and electrical pathways toward ultrasensitive spin readout.

This Account describes our progress toward the discovery and realization of mechanisms that allow for the ultrafast preparation of pure magnetic states through SF and the development of new tools and techniques to understand and ultimately deploy these systems as a quantum technology. In particular, we have shown that molecular symmetries can be exploited to generate state-specific preparation and that molecular structures with rigid architectures can minimize the fluctuations that drive spin decoherence. Both effects lead to coherence times that are orders of magnitude longer than the “gate switching” time at temperatures much higher than the operating temperatures in superconducting quantum hardware.

## 2 Relaxation Theory for the Biexciton and Time-Resolved Electron Paramagnetic Resonance Spectra

Singlet fission is an ultrafast internal conversion that transforms an optically bright singlet state into two dark triplet states through a biexciton intermediate that initially retains singlet spin multiplicity.^26^ While its initial discovery came from fundamental research into the photophysics of molecular solids,^27^ a resurgence in activity has stemmed from its potential utility in photoconversion.^28^ We leverage a subset of this research that has uncovered properties associated with the SF intermediate, the triplet pair.^29−32^ Here, we use a frontier molecular orbital picture for two chromophores, *A* and *B*, that can host a spin singlet or spin triplet Frenkel exciton. Such a simple picture is commonplace in the singlet fission literature, but it relies on many assumptions that will not be repeated here.^26^ The ground state absorbs a photon and enters a singlet state localized on one chromophore (S_0_S_*n*_), which then undergoes interconversion to a singlet biexciton state ^1^TT, a linear combination of the two triplet excitons on each chromophore. Once in the ^1^TT state, there is a slower spin nonconserving intersystem crossing process from ^1^TT to other spin superpositions of the biexciton, ^1^TT → ^2*S*+1^TT_*M*_, with multiplicity 2*S* + 1 and a magnetic quantum number for the sublevels *M* ∈ −*S*,···0,···*S*. The products of intersystem crossing are detectable in time-resolved electron paramagnetic resonance (trEPR) experiments that detect magnetic dipole transitions with selection rules Δ*S* = 0 and Δ*M* = ± 1. The objective of the theory is to explain how the ^1^TT state undergoes intersystem crossing, how the products appear in the trEPR spectra, and how both the inter- and intramolecular structure dictate the excited-state trajectory using a simple model with as few parameters as possible.

### 2.1 The JDE Hamiltonian

We do not recapitulate the full physical reasoning behind our approach here but instead summarize our results and the experimentally testable predictions that flow from it. Readers interested in the detailed development of our model are referred to our original work^1,33,34^ and related formulations.^35,36^ The spin Hamiltonian for a pair of chromophores in a magnetic field is parametrized by the intermolecular exchange (*J*), the intramolecular dipole coupling (*D*, *E*) and the intermolecular anisotropic (*X*) interactions; thus, we call it the JDE(X) Hamiltonian.
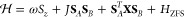
1While our derivation of the JDE Hamiltonian started in the “all-electron” picture, it is a function of the exciton spins **S**_*A*_ and **S**_*B*_. For *S*_*A*_ = *S*_*B*_ = 1, there are only 9 states that one must consider—far fewer than the number of Slater determinants in the frontier molecular orbital picture. The JDE Hamiltonian describes how two spin excitons in a magnetic field interact with both one another and themselves.

The first term in eq 1 is the Zeeman Hamiltonian in EPR experiments that splits the *M* sublevels, where *S*_*z*_ is the *z*-projection of the total angular momentum, **S** = **S**_*A*_ + **S**_*B*_. The frequency ω is proportional to the static field *B*_0_ that is swept to find resonances in X-band EPR experiments. The second term is the isotropic, or rotationally invariant, Dirac–Heisenberg exchange coupling between exciton spins. Both of these terms are commute with **S**^2^. They are both diagonal in the total spin basis |*S*, *M*⟩, where the quantization axis lies along the direction of the Zeeman field.

The Hamiltonian in eq 1 is compact and physical, but it is not irreducible. To apply rigid body rotations, we symmetrize the Hamiltonian by writing its operators in terms of irreducible spherical tensors. Once transformed, the orientationally dependent selection rules for the ^1^TT state into the ^2*S*+1^TT_*M*_ states follow the Wigner–Eckart theorem.

Besides the fact that the |*S*, *M*⟩ basis transforms symmetrically under rigid body rotations, it is convenient for expressing the Hamiltonian for several reasons. First, the initial biexciton state is an eigenstate of |*S*, *M*⟩, with |*S* = 0, *M* = 0⟩. Second, the EPR experiment induces transitions between different *M* sublevels within the same *S*, so interpretations of the spectra are simple on this basis. Lastly, for the molecules that we study in X-band EPR, the first two terms are the largest, which means that away from degeneracies, the **X**-interaction and the zero-field Hamiltonian are perturbatively weak in the total spin basis away from level crossings.

### 2.2 Dynamics in the JDE Hamiltonian

The initial ^1^TT state is an eigenstate of the total spin but not of the total Hamiltonian. It is, therefore, a nonstationary state that will evolve in time. The mechanisms for its time evolution depend sensitively on the system. If *J* is weak, the diabats can mix strongly so that the dynamically relevant states are the adiabats. The ^1^TT state then evolves along the adiabatic states and undergoes “curve-crossing” dynamics that are reminiscent of adiabatic energy and electron transfer mechanisms.

**Figure 2:**
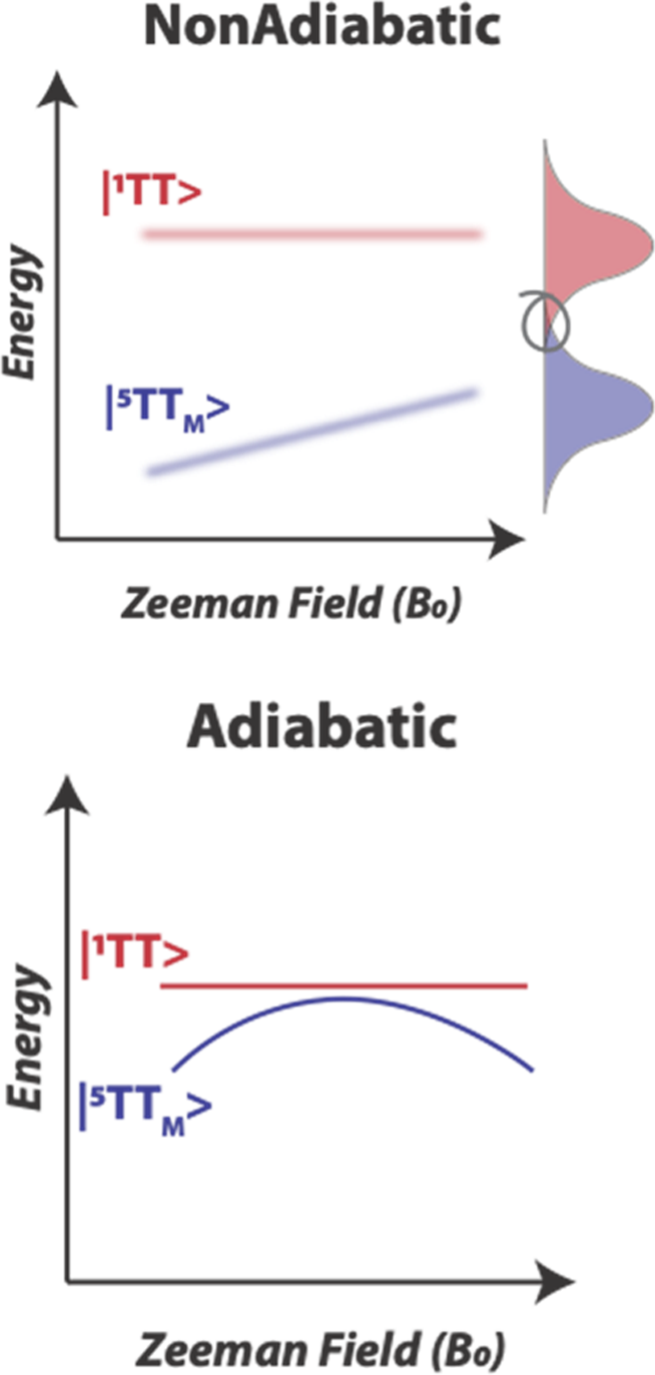
Nonadiabatic (top) and adiabatic (bottom) mechanisms for state-to-state transitions. In the nonadiabatic mechanism, energy levels (blurred lines) are separated by large gaps. The red and blue curves denote the fluctuation spectrum of the levels, which are the labeled diabats. While the gaps are large on average, nuclear fluctuations occasionally bring the two levels into resonance (gray circle) and allow transitions to take place. In the adiabatic mechanism, energy gaps are small. Two energy levels hybridize in the vicinity of a crossing (avoided crossing depicted here), allowing the transition to occur. Away from the crossing, the adiabats and diabats coincide. Our analysis of the JDE model employs nonadiabatic transition theory.

We have posited that the relaxation mechanism is different in the systems that we have studied, driven not by gentle curve crossings between adiabats but by sudden nonadiabatic transitions between states that are mostly diabatic (Figure 2). While the first two terms in eq 1 conserve the total spin, the other terms do not. As the exciton pair moves, either though nuclear fluctuations or exciton hopping, the JDE Hamiltonian becomes time-dependent.

Specifically, we imagine that *J* is a time-dependent parameter whose mean value ⟨*J*⟩ is larger than the next largest energy scale, *D*. Nuclear fluctuations cause significant and large deviations from equilibrium such that *J*(*t*) = ⟨*J*⟩ + *δJ*(*t*). In the basis of total spin, |*S*, *M*⟩, the Hamiltonian with the chromophores in a specific orientation can be written in a set of sectors of total *S*. Within each sector, there are couplings between *M* sublevels that may be included by diagonalizing the sector and redefining the Hamiltonian in this “adiabatic” basis or by ignoring them and choosing the diabatic basis. The adiabatic choice gives trEPR spectra that match experiments more closely, while the diabatic choice facilitates interpretation. In the limit |⟨*J*⟩/*D*| ≫ 1, states of various *M* mix weakly, and the differences between these two choices are usually small for most molecular orientations that we have explored.

To simplify the discussion, we use the diabatic choice. The first event is the transition from ^1^TT to states of ^5^TT_*M*_. This occurs through a nonadiabatic transition with a rate proportional to that of the tunneling matrix element |⟨^1^TT|*H*_ZFS_|^5^TT_*M*_⟩|^2^. Spin polarization is the result of the selection rules after the application of the Wigner–Eckart theorem. These selection rules are weaker for *M* than they are for *S*. In particular, for the symmetric chromophore geometries that we have studied, two states with spin quantum numbers *S* and *S*′ must satisfy *S* + *S*′ ≥ 2, and if the chromophores are all parallel, both *S* and *S*′ must be even. The result is that the triplet states ^3^TT_*M*_ can only be populated from the quintet, and if all chromophores are parallel, they are removed from the manifold entirely.

### 2.3 The JDE Criteria

The calculations present three clear and specific design rules for creating molecular assemblies that drive the excited-state population exclusively to the ^5^TT_0_ state. We refer to these as the JDE criteria:

1. Interchromophore exchange interactions (*J*) should be strong enough to split the ^*n*^TT levels and avoid curve crossings at the experimentally relevant magnetic field.^2^ At a minimum, *J* must be larger than *D*, the magnetic dipole coupling that splits the magnetic sublevels of each spin multiplicity.
2. One should limit triplet exciton diffusion because it unpairs the biexciton and generates incoherent triplets.
3. Chromophores should be oriented with their principle molecular axis parallel to the applied magnetic field and to each other to enforce the desired selection rules.

## 3 Triplet-Pair Systems that Test Model Predictions

### 3.1 Covalent Dimers

Covalent dimers have served as important architectures in the interrogation of singlet fission, particularly for studying the evolution of the bright single excitation state into the multiexciton manifold. A number of platforms have been explored ranging from bis-diimides^37^ to bisacenes^38,39^ with a number of groups and dimer systems contributing beyond what we are citing here. In this same context, covalent dimers have been valuable for exposing how EPR-active multiexciton states such as ^5^TT are generated and how lineshapes and spin interconversion dynamics relate to the structural details imposed by the synthetic strategies that are used.^40^ These versatile systems also provide one clear route to achieve a subset of the JDE criteria.^3,41^ Such molecular assemblies can allow for the design of the isotropic coupling between triplet spin centers and anisotropic triplet/triplet interactions that depend on the relative arrangement of chromophores. The comprehensive alignment of such dimers with respect to an applied magnetic field, either on surfaces or in crystals, remains a challenge, and here, we report our achievements studying isotropic glasses.

**Figure 3:**
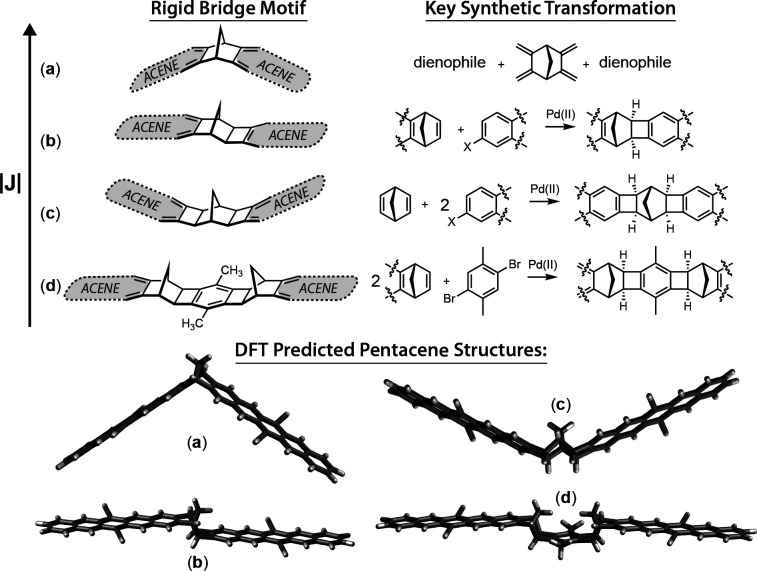
Top: different bridge motifs and synthetic approaches. Bottom: computational models of acetylene-substituted pentacene dimers.

Unlike most prior covalent dimer investigations, to completely control the molecular frame, we have sought rigid bridges with multiple points of attachment to each of the two chromophores. Such a paradigm is generally valuable for studying SF by way of removing the structural and dynamical heterogeneity impacting spectroscopic observations.^42^ We have developed methods for preparing tetracene, pentacene, and anthracene dimers with rigid bridges.^43−45^ Some motifs are shown in Figure 3, including a bicyclic norbornyl spacer (**a**) that has been used extensively^42−44^ as well as newer systems that also incorporate cyclobutene fragments in various ways (**b**–**d**).^45^ This motif was inspired by seminal energy and electron transfer research by Paddon-Row and co-workers, who called upon bicyclic fragments and related derivatives to control the spatial juxtaposition of donor and acceptor moieties.^46,47^ In the first dimer system (**a**), the two chromophores housing the spin centers in the biexciton state are not fully coplanar but instead share one common magnetic axis (the short axis of each chromophore). We used this platform in a pentacene dimer called TIPS-BP1′ to explore both spin polarization and coherence in the ^5^[TT] manifold.^3^ This was our starting place, as we had a firm understanding of the optical dynamics,^42^ and the smaller bridge was expected to provide a situation where *J* > *D*. *J* represents the exchange coupling between spin centers located on the two respective chromophores, while *D* involves the dipolar interactions between the unpaired electrons on an individual chromophore (the only meaningful terms). Since *J* varies exponentially with interchromophore distance and *D* varies based on the geometry of each chromophore, it is anticipated that at short distances, increases in *J* exceed *D*. The other bridge motifs modify *J* in systematic ways while providing viable routes in two of the cases (**b** and **d**) toward more coplanar arrangements of chromophores.

**Figure 4:**
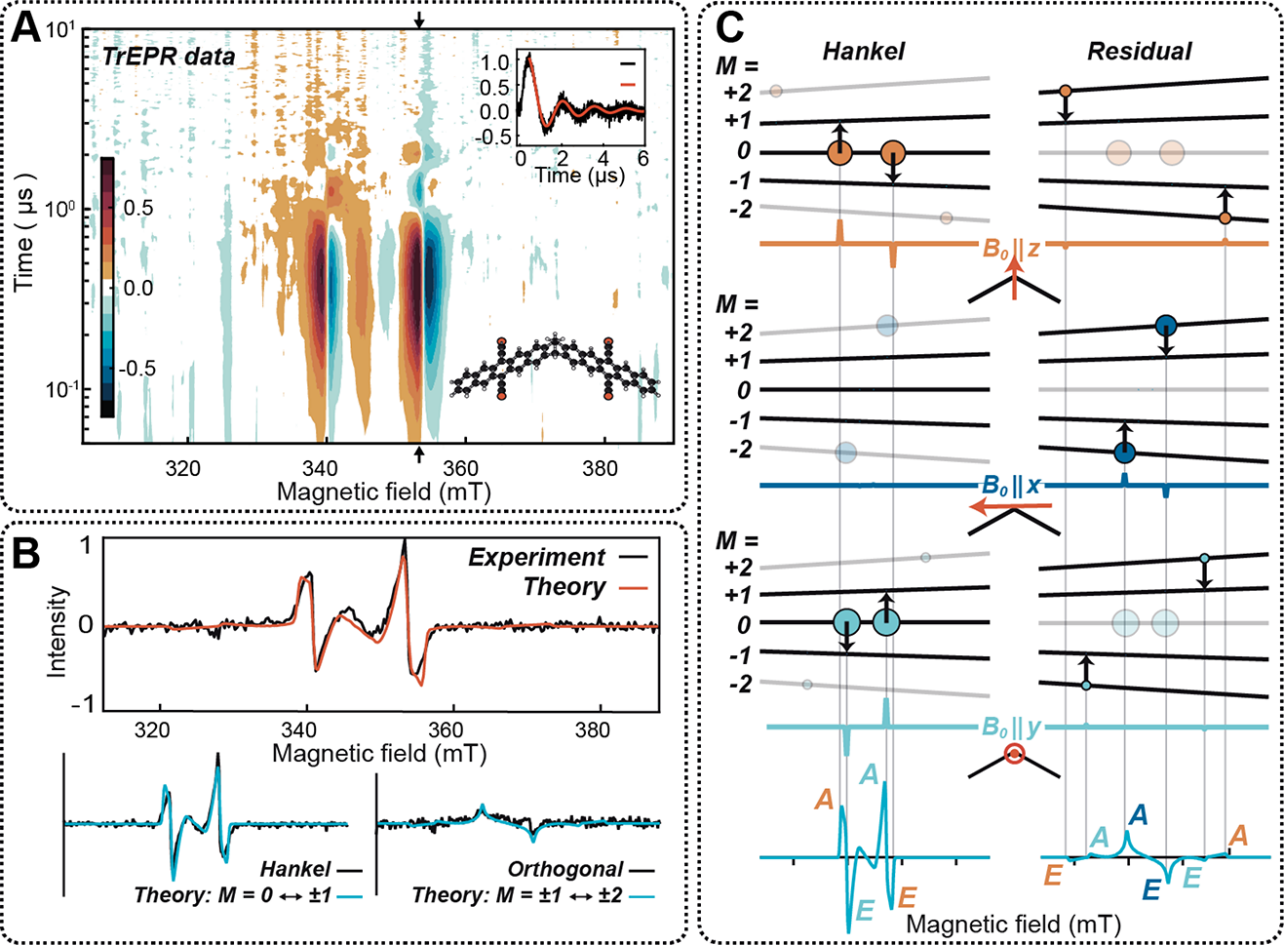
(A) Contour plot of X-band trEPR data for TIPS-BP1′ (75 K and 640 nm excitation). (B) The prompt trEPR spectrum for TIPS-BP1′ (black) is an average over 200–400 ns (from panel A). The red line comes from the JDE model. (C) Fixed-orientation EPR spectra (colored lines) for *B*_0_ (red arrows) applied along cardinal dimer directions. Predictions for the Hankel spectrum (^5^TT_0_ ↔ ^5^TT_±1_) are on the left, and those for the residual spectrum (^5^TT_±1_ ↔^5^TT_±2_) are on the right. Area of colored circles indicates ^5^TT_*M*_ sublevel populations. Vertical lines correlate population assignments with features in the simulated powder spectra (light blue, below). Reproduced with permission from ref (3). Copyright 2023 The Authors. Published by Springer Nature under a Creative Commons Attribution 4.0 International License.

trEPR measurements were made for TIPS-BP1′ following pulsed visible photoexcitation (see Figure 4A). The field-swept spectra reveal both absorptive (positive) and emissive (negative) transitions between magnetic sublevels, as magnetically active species are born and evolve out of the optically prepared singlet (S_1_ and ^1^TT) species. Like other dimer studies in the literature, the absorptive and emissive features that are first observed are those whose narrow distribution heralds the formation of ^5^TT. Indeed, the prompt spectrum (averaged over 200–400 ns) shown at the top of Figure 4B is cleanly modeled as a quintet using the JDE theory with only three best-fit parameters, corresponding to the zero-field splitting parameter *D*, an anisotropic exchange interaction between the *S* = 1 spin centers X, and the angle between the two chromophore π faces (called β). Remarkably, the best fit suggests that β = 111.1 ± 0.2°, which is in near perfect agreement with density functional theory (DFT) modeling.

One of the salient features in the data is the manifestation of Rabi oscillations as a function of time, indicating that the strong-field microwave radiation drives population inversion among magnetic sublevels prepared coherently following photoexcitation. A simple but important consequence is a direct measure of the decoherence time in this system, which we find to be 1.4 μs, even at the elevated (77 K) temperature of the experiment. This is a time scale that is orders of magnitude longer than the switching times needed for gate operations in quantum devices. A more nuanced but valuable analysis involves the application of a Hankel transform, a procedure directly analogous to a Fourier transform. Rather than being composed of a series of sinusoids (the Fourier series), the Hankel transform uses the Bessel series of the first kind, which can be intuitively understood as exponentially decaying sinusoids. It is thus a natural way of representing trEPR spectra that exhibit decaying Rabi oscillations. The Hankel spectrum, then, is the spectral distribution of a component that oscillates at a specific frequency, allowing for the isolation of specific spin species by their Rabi frequency. JDE theory nearly quantitatively predicts these spectra in both their functional form and their amplitude: the Hankel spectrum is derived from ^5^TT_0_ → ^5^TT_±1_ transitions, while the residual spectrum comes from small populations originating in ^5^TT_±2_ (Figure 4B). A final point is that the magnetic sublevel populations that are produced following the photoexcitation of TIPS-BP1′ are intimately tied to the orientation of the dimer and its two sets of magnetic axes relative to the laboratory frame magnetic field, *B*_0_. The agreement between theory and experiment thus shows us ideal orientations if conditions of state purity are of paramount importance. For example, as shown in the bottom case in Figure 4C, one would strive to prepare an ensemble of dimers where the parallel short axes of the two chromophores are aligned with *B*_0_.^3^

### 3.2 Crystals

Molecular crystals offer an opportunity to explore a different subset of the JDE criteria from that found in covalent dimers, as they are readily aligned with respect to an external magnetic field and provide definite packing arrangements. A growing number of reports show evidence for quintet formation in (poly)crystalline samples,^30,38^ but it is a significant challenge to engineer systems that exhibit truly dimer-like interactions with no opportunity for TT state dissociation into T + T. Here, we explore systems that approach this ideal.

Our trialkylsilyl-based functionalization scheme provides a tunable, parallel, and cofacial alignment of chromophores in crystals.^49^ For example, in pentadithiophenes (PDTs), variation in the size of the trialkylsilyl group shifts interactions from a strongly coupled two-dimensional (2D) “brickwork” crystal packing to a more weakly coupled one-dimensional (1D) “slipped stack” arrangement. To further weaken intermolecular interactions, we found that the end-substitution of the acene was critical. For example, the addition of alkyldioxolane groups to the end of pentacene led to chromophores that maintained their coplanar, coaxial orientation but had separation of the π faces by more than 5 Å, allowing for solid-state fluorescence.^50^ In PDTs, adding terminal triisopropylsilyl groups led to a further weakening of the electronic coupling between molecules, and the chromophores adopted only a roughly coaxial arrangement.^25^

**Figure 5:**
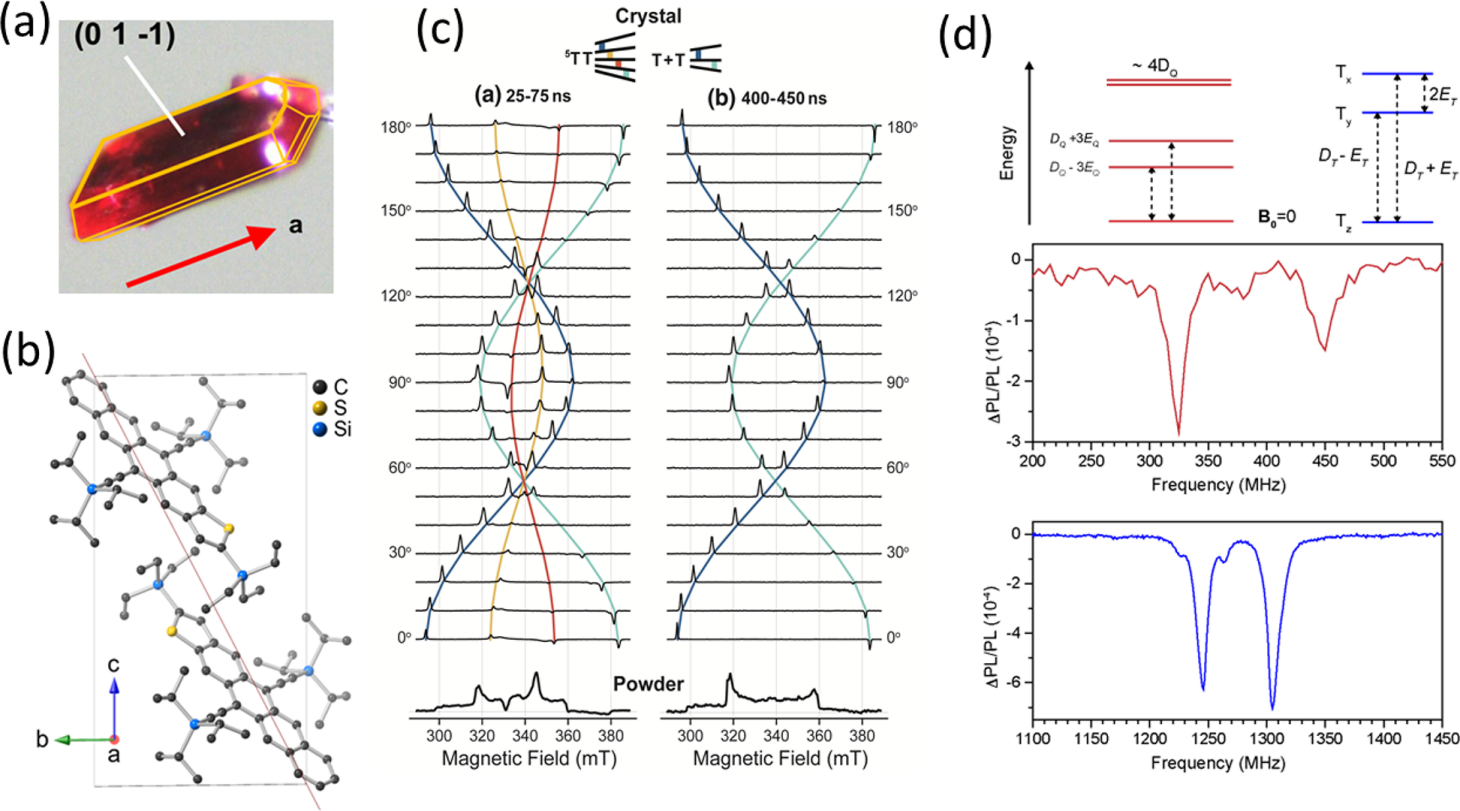
(a) Indexed crystal of TES TIPS TT and (b) unit cell structure. (c) Crystal-angle-dependent trEPR spectra at early and late delay times, showing quintet and triplet transitions that reveal the population flow, as it depends on molecular axis alignment with the magnetic field. Simulations based on the JDE model are shown as solid lines for the transitions color-coded in the spin manifolds at the top. The bottom panel shows associated powder spectra. Adapted with permission from ref (2). Copyright 2022 The Authors. Published by PNAS under a Creative Commons Attribution-NonCommercial-NoDerivatives License 4.0 (CC BY-NC-ND). (d) Predicted transitions within ^5^TT (red) and T_1_ (blue) manifolds, and the zero-field PLDMR spectra. Adapted with permission from ref (4). Copyright 2022 The Authors. Published by AIP Publishing under a Creative Commons CC BY 4.0 DEED Attribution 4.0 International License.

For tests of the JDE model, we desired a local, pairwise interaction of chromophores, which we have found is most easily induced by reducing the symmetry of the chromophore. Rather than using our usual tactic of moving the attachment position of the silyl-based solubilizing group,^51,52^ we instead converted one terminal benzene ring on the chromophore to a thiophene, which allowed us to attach a sterically demanding triethylsilyl group at that point. This desymmetrization induced coplanar, coaxial, pairwise stacking of the thienotetracene chromophore, and these π-stacked pairs then arranged so that the solubilizing substituents of one stack overlapped adjacent stacks, effectively isolating the dimeric pairs (Figure 5b).

### 3.3 TrEPR of an Oriented Single Crystal

The aforementioned material 2-triethylsilyl-5,11-bis(triisopropylsilyl ethynyl) tetraceno[2,3-b]thiophene (TES TIPS TT) can be crystallized in large monoliths (Figure 5a), which can be indexed and then oriented with respect to a laboratory axis (i.e., static magnetic field direction **B**_0_). This allows EPR experiments to be performed as a function of the angle between the principal magnetic directions of the molecular frame, defined by molecular symmetry, and **B**_0_ in the EPR cavity. This provides a powerful test of the JDE model.

Experiments that detected emission versus the magnetic field strength show crossings between ^1^TT and ^5^TT at field positions that reveal the value of *J* to be roughly 15 GHz, which is safely in the *J* > *D* regime (*D* has been found to be roughly 1.2 GHz).^2^ The JDE model predictions of the peak position shown in Figure 5c track the experimentally observed shifting that is characteristic of a fully aligned sample oriented along different field directions. Further, the populations in the ^5^TT sublevels follow a dependence that is also predicted from theory: as the principal molecular axis *z* is tuned away from **B**_0_, the populations move from strictly ^5^TT_0_ toward ^5^TT_±2_. Free triplets T + T are also predicted to be spin-polarized depending on whether they are consanguineous. Therefore, T + T resulting from ^5^TT_0_ at **B**_0_ parallel to *z* are solely T_0_-polarized with further contributions from T_1_ as other ^5^TT spin sublevels are available as precursors. The value of *J* also allows the prediction of magnetic sublevel crossings that facilitate population flow between the ^3^TT and ^5^TT states, leading to trEPR features at X-band magnetic fields (e.g., at 100° in Figure 5c) that do not obey the symmetric peak pattern typically observed for triplet pairs formed away from curve crossings.

The observation of T + T in TES TIPS TT crystals suggests that intermolecular coupling outside of the primary dimer that performs SF allows triplet transport, which reduces exchange into the weak coupling limit. These “free” triplets retain spin memory initially and may be useful in some contexts, but they may also be subject to undesirable decoherence, as they sample the crystal environment through hopping. Broad features in the trEPR spectra at early times also suggest that more than one type of quintet is formed and may be in fast equilibrium with other triplet-pair sites, an outcome that violates the original goal of a single species initialized in a pure state. These observations lie outside the JDE model predictions, which otherwise provide excellent agreement with the experimental results and suggest that strict constraints toward isolated dimer-like interactions are crucial.

## 4 Population-Detected Magnetic Resonance

In addition to trEPR, optically or electrically addressed magnetic resonances can provide both insight and utility. By using electronic state concentrations, these “population”-detected magentic resonance methods offer greater specificity and/or sensitivity than can be achieved through microwave absorption. We will describe three efforts in this space to utilize and advance the state-of-the-art understanding of optically initialized triplet spin polarization and its evolution. Photoluminescence and electrical readout mechanisms offer a route toward single-molecule sensitivity, while photoinduced absorption-detected magnetic resonance proves to be a versatile tool for correlating optical and magnetic excited-state spectra.

### 4.1 Photoluminescence-Detected Magnetic Resonance (PLDMR)

Photoluminescence (PL)-detected magnetic resonance has often been used to characterize how emission depends on the spin sublevel population. The classic example in emerging quantum systems is the nitrogen vacancy in diamond.^53^ For *S* = 2 quintets, selection rules would suggest dark character, but the reversibility of ^1^TT–^5^TT transitions may modulate the population of singlet character bright states and, thus, the overall emissivity. In particular, the spin sublevel ^5^TT_0_ may have a stronger kinetic pathway to bright levels compared to other quintet sublevels. Thus, PLDMR could provide a highly sensitive mechanism of spin-state readout, especially given the selectivity toward ^5^TT_0_ conferred by adherence to the JDE criteria.

TES TIPS TT was found to be moderately emissive with a spectral shape that transformed at low temperatures to resemble that of other ^1^TT emitters.^54^ We were able to demonstrate a strong spin memory effect: at low excitation densities, the ^1^TT emission was modulated by magnetic resonance transitions characteristic of both uncoupled T_1_ and ^5^TT states, the former of which remained spin-correlated due to their common origin in ^1^TT (Figure 5d). Increasing the triplet concentration (light intensity) changes the mode of recombination through ^1^TT from geminate to nongeminate, and the spin memory is lost.^4^ However, these results show that under the right conditions, an equilibrium between ^1^TT and higher spin states can be exploited for sensitive spin readout.

### 4.2 Magnetoconductance

The idea of employing an electrical current to read out triplet exciton spin is inspired by the Pauli blockade principle^55^ that underpins the sensitive spin readout mechanism in lithographically defined quantum dots.^56,57^

**Figure 6:**
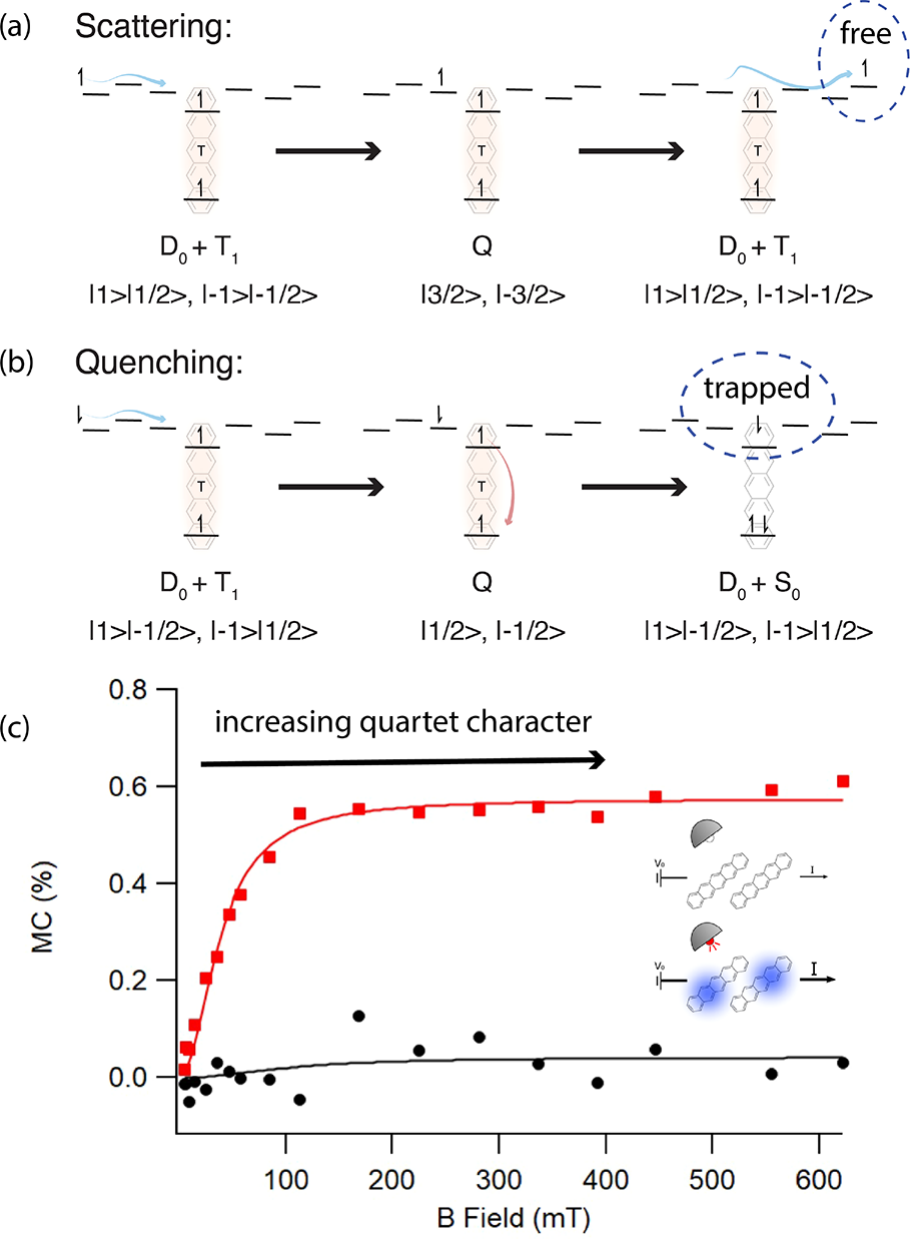
(a, b) Diagrams of the hypothesized mechanism of magnetoconductance in single-carrier pentacene devices, showing spin configurations that lead to scattering via (a) the high-spin quartet configuration versus (b) quenching in the doublet or low-spin quartet configuration, all in the high-field doublet, triplet, and quartet bases, as indicated in the figure annotations. When triplets occupy charge trap sites, scattering via the high-spin quartet configuration leads to increased conductivity because the trap remains filled. Quenching of the triplet by the charge empties the site, allowing for the trapping of charge carriers, thus reducing the conductivity. Electron transport is illustrated for diagrammatic simplicity, but equivalent physics applies to holes. (c) Magnetoconductance measurements on hole-only polycrystalline pentacene diodes. The red symbols are illuminated to generate triplet excitons via singlet fission, while the black symbols are measured in the dark. The solid lines show fits to the MC data using a kinetic model that implements the hypothesis outlined in (a, b). Adapted with permission from ref (24). Copyright 2022 American Chemical Society.

First, however, we must understand the interactions between the ground-state doublet associated with charge carriers on organic molecules and proximal triplet excitons. The basic theory of the triplet–doublet interaction is quite simple. When a triplet exciton and a doublet charge collide, there are six possible spin configurations. At a zero applied magnetic field, all of them have a projection onto the doublet ground state (S_0_ + D_0_), making quenching of the triplet spin permissible.^58,59^ However, as the magnetic field increases, this system resolves into a doublet and quartet manifold, and the *m*_s_ = ±3/2 states of the quartet have no overlap with the doublet, which forbids quenching for those configurations and thus decreases the overall rate. These spin statistics are illustrated in Figure 6a,b using the high-field uncoupled doublet–triplet basis and quartet basis, as appropriate. Both of these possible outcomes (quenching and scattering) might influence current in their own way. Our work has shown that such behavior dominates the magnetoconductance behavior of bulk pentacene devices, making it a potentially useful mechanism for the readout and control of triplet excitons.^24^

The key result is shown in Figure 6c, giving the magnetoconductance curves in the light (red) and dark (black) regions, which were fit using our quantum kinetic model. The quartet character of the triplet–doublet encounter complex is calculated via the stochastic Liouville equation (SLE), and the magnetoconductance is modeled by solving a system of kinetic equations at steady-state with the SLE populations as an input parameter.^24^ We invoke a trap filling mechanism to explain this counterintuitive increase in conductance with the magnetic field, illustrated in Figure 6a,b. Trap sites for charge carriers are correlated with those for triplet excitons, and at high exciton densities, when the applied magnetic field has minimized exciton charge quenching, they become filled with triplets.

### 4.3 Photoinduced Absorption-Detected Magnetic Resonance (PADMR)

A vexing problem with studying spin manifold evolution in singlet fission systems is that spin-state evolution can be very rapid compared to the time resolution of standard trEPR spectroscopy, and not all of the relevant states exhibit magnetic susceptibility, such as ^1^TT. This often motivates the use of optical transient absorption methods, but here the problem is that it can be extremely difficult to distinguish different triplet-pair species from differences in their optical spectra. A new tool is needed to bridge the gap between optical and magnetic spectroscopy. To this end, we developed a novel implementation of photoinduced absorption-detected magnetic resonance^23^ and applied it to understand the transient optical spectrum of a parallel-pair crystalline material, tri-2-pentylsilylethynyl pentadithiophene (TSPS-PDT).^25^ Like TES TIPS TT, described in the preceding section,^4^ TSPS-PDT was designed to form slip-stacked crystals with parallel principal molecular axes and minimal intermolecular coupling to inhibit ^*n*^TT dissociation into consanguineous T + T states and to funnel the population toward ^5^TT_0_, in accordance with the JDE model.

**Figure 7:**
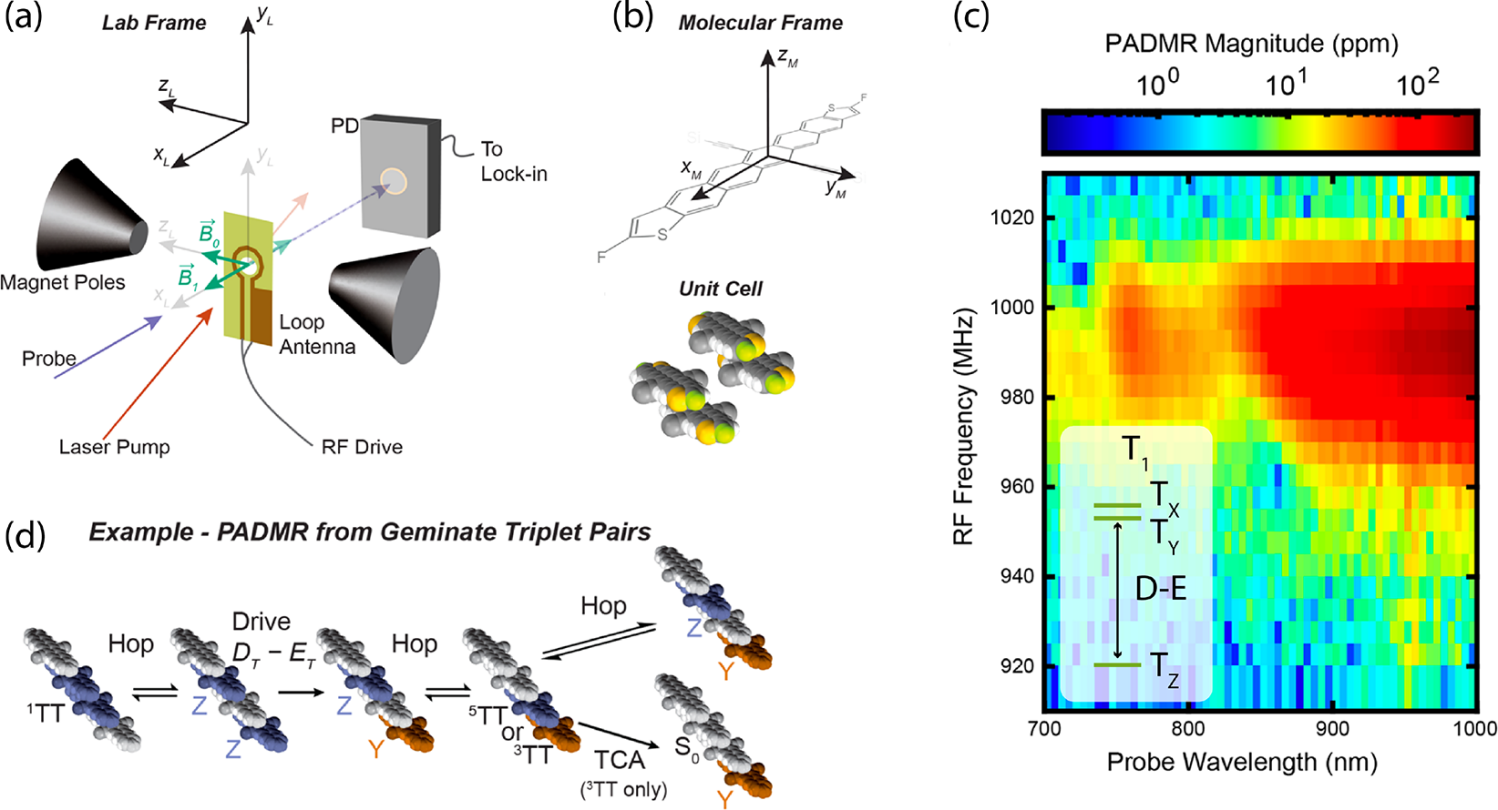
(a) Diagram of the functional configuration of our PADMR instrument. (b) The molecular structure and principal axes of TSPS-PDT, along with a representation of the crystalline unit cell. (c) The correlation map between drive frequency and the optical probe wavelength in the NIR region, centered on the D–E transition within the T_1_ manifold, as shown in the inset energy level diagram. (d) A proposed kinetic scheme that accounts for the spectrum in (c) as arising from kinetic coupling between ^1^TT and a spin-correlated T + T state. Driving the D–E transition near 990 MHz in the T_1_ manifold forbids fusion back to ^1^TT, thus depleting the ^1^TT concentration at steady-state and giving the negative NIR PADMR spectrum that we observe in (c). Adapted with permission from ref (23). Copyright 2023 The Authors. Published by American Chemical Society under a Creative Commons CC-BY 4.0 License.

Figure 7a shows a diagram of our instrument, and Figure 7b shows the staircase-like crystal structure of TSPS-PDT. The sample, in this case a polycrystalline thin film, is deposited on a sapphire substrate and positioned directly over a microstrip or a coplanar waveguide coupling loop with a hole bored through the center to allow the probe light to pass through. Figure 7c shows the main result of this work: a correlation map of the response of the near-infrared (NIR) photoinduced absorption between 700 and 1000 nm in response to an radio frequency (RF) drive between 910 and 1030 MHz at a zero applied magnetic field. The 2D spectrum is displayed as the log of the PADMR signal magnitude, which turns out to be negative in sign. Moreover, our analysis of the total f-PADMR (microwave frequency-swept) spectrum detected at visible wavelengths reveals that the transition peaking at 990 MHz is associated with T_1_ states, not ^5^TT states, which is just barely detectable in this system at 350 and 1200 MHz.^23^

Prior work has assigned the NIR photoinduced absorption to ^*n*^TT states largely on the basis that they do not appear when these molecules are sensitized with triplet states in solution, and they are kinetically correlated with the visible (600 nm) feature that is associated with the total triplet population. Figure 7 illustrates our hypothesis. We propose that in TSPS-PDT, these NIR absorption features are dominated by a large population of ^1^TT, and we are able to detect this magnetically dark singlet species in the PADMR experiment due to a kinetic coupling to geminate T + T species.^23^

A remarkable implication of this observation is that, similar to the low-flux case in PLDMR of TES TIPS TT, T + T remains appreciably spin-correlated into the steady-state time scale of this experiment (hundreds of microseconds). While the main thrust of our work has been focused on understanding and preparing a pure magnetic sublevel population of ^5^TT, these results highlight a different potential quantum resource: separated triplets that retain the spin memory of their formation, perhaps to the extent that they are an entangled pair.

## 5 Conclusions and Outlook

The body of work described herein shows that SF can be an effective route to prepare pure magnetic states at elevated temperatures and that this behavior can be controlled through molecular engineering. We developed a theoretical description of SF dynamics that agrees with multiple different experiments on both covalently bound dimers and single crystals of various polyacene materials. This work can be boiled down to a surprisingly simple set of design rules for SF systems that populate only ^5^TT_0_ after photoexcitation, which we refer to as the JDE criteria. Our work implementing novel spin-sensitive spectroscopy tools has allowed a more complete experimental understanding of SF dynamics and ultimately points the way toward a future of single-molecule sensitivity via electrical- or luminescence-based spin readout. This lays the groundwork for future experiments that might use these pure magnetic states to initialize or operate on a register of proximal ground-state spins (nuclear or electronic) to enable unique quantum sensing or computing elements that operate at high temperatures and are tailored to the application via the well-developed power of synthetic organic chemistry.
